# Bimetallic MOFs-Derived Hollow Carbon Spheres Assembled by Sheets for Sodium-Ion Batteries

**DOI:** 10.3390/nano12213926

**Published:** 2022-11-07

**Authors:** Hui Yang, Ang Li, Chunli Zhou, Xuewei Liu, Xiaohong Chen, Haiyan Liu, Tao Liu, Huaihe Song

**Affiliations:** 1 State Key Laboratory of Chemical Resources Engineering, Beijing Key Laboratory of Electrochemical Process and Technology for Materials, Beijing University of Chemical Technology, Beijing 100029, China; 2 Tangshan Key Laboratory of Optoelectronic Conversion Materials, School of Physical Science and Technology, Tangshan Normal University, Tangshan 063000, China; 3 Shandong Energy Group Ltd., Zhoucheng 277527, China

**Keywords:** bimetallic-MOFs, p-phenylenediamine, hollow carbon spheres assembled by sheets, sodium-ion batteries

## Abstract

Metal-organic frameworks (MOFs) have attracted extensive attention as precursors for the preparation of carbon-based materials due to their highly controllable composition, structure, and pore size distribution. However, there are few reports of MOFs using p-phenylenediamine (pPD) as the organic ligand. In this work, we report the preparation of a bimetallic MOF (CoCu-pPD) with pPD as the organic ligand, and its derived hollow carbon spheres (BMHCS). CoCu-pPD exhibits a hollow spherical structure assembled by nanosheets. BMHCS inherits the unique hollow spherical structure of CoCu-pPD, which also shows a large specific surface area and heteroatom doping. When using as the anode of sodium-ion batteries (SIBs), BMHCS exhibits excellent cycling stability (the capacity of 306 mA h g^−1^ after 300 cycles at a current density of 1 A g^−1^ and the capacity retention rate of 90%) and rate capability (the sodium storage capacity of 240 mA h g^−1^ at 5 A g^−1^). This work not only provides a strategy for the preparation of pPD-based bimetallic-MOFs, but also enhances the thermal stability of the pPD-based MOFs. In addition, this work also offers a new case for the morphology control of assembled carbon materials and has achieved excellent performance in the field of SIBs.

## 1. Introduction

In recent years, with the wide application and rapid development of portable electronic devices and electric vehicles, the demand for secondary batteries has gradually increased, and the green renewable resources have become a research hotspot [1]. The high cost and shortage of lithium resources limit the large-scale application of lithium-ion batteries (LIBs) [2,3]. As an alternative, sodium-ion batteries (SIBs) will be more competitive in the future large-scale energy storage market due to their abundant sodium resources, low cost, similar working principles to LIBs, high safety performance, and wide operating temperature range [4]. However, the relatively large size of sodium ions also poses a great challenge to the structural stability of sodium storage materials, and will lead to slow electrochemical reaction kinetics, affecting the sodium storage capacity and rate performance [5,6]. Therefore, the design and preparation of electrode materials is the key to promote the development of SIBs. As an important part of SIBs, the anode material has an important influence on the performance of SIBs [7]. To date, the reported anode materials have mainly focused on carbon-based materials, alloy-based materials, and metal compounds [8]. Among them, carbon-based materials have good cycle stability, electrical conductivity, abundant raw materials, and simple preparation methods, making them a standout candidate for SIBs anode materials [9].

MOFs have the advantages of multiple designability (structure, composition, pore size) and a large specific surface area [10], and are considered as good precursors and templates for the preparation of carbon materials with tunable structures and high specific surface areas [11,12]. The MOFs-derived carbon material inherits the advantages of the MOF precursor while enhancing its poor electrical conductivity and improving the abundant reaction sites; MOFs-derived carbon materials are widely regarded as a new material with excellent electrochemical activity and have attracted much attention in the field of sodium storage [13]. Xu et al., synthesized a series of metal-organic frameworks (MOFs) using terephthalic acid as the organic ligand, and prepared selenide/carbon composites by pyrolysis as anode materials for SIBs. Among them, the uniform peapod-like Fe_7_Se_8_@C nanorods have a high specific capacity (218 mA h g^−1^ after 500 cycles at 3 A g^−1^) and the porous NiSe@C spheres still have a specific capacity of 160 mA h g^−1^ after 2000 cycles at the current density of 3 A g^−1^ [14]. Ge et al., prepared MOFs-derived core/shell structured CoP@C polyhedrons anchored on 3D reduced graphene oxide networks for SIBs, which exhibited excellent cycling stability and high rate performance [15]. Zou et al., synthesized Fe-MOF with Fe(NO_3_)_3_·9H_2_O and fumaric acid, and obtained rod-like Fe_7_S_8_/C composites by vulcanization, which showed a specific capacity of about 500 mA h g^−1^ after 100 cycles at a current density of 0.1 A g^−1^ [16].

In recent years, more and more MOFs-derived carbon materials have been used as anode materials for SIBs, but the organic ligands of MOFs are mainly concentrated in imidazoles and carboxylic acids. There are few reports of MOFs with p-phenylenediamine (pPD) as the organic ligand, which may be related to the unstable skeleton structure and the inability to maintain the morphology during pyrolysis [17]. Many studies have shown that, compared with monometallic MOFs, bimetallic MOFs contain two metal active sites, which can not only tune the morphology and structure of MOFs, but also increase the complexity of the structure where the structures support each other during the pyrolysis process, which is beneficial to improve the stability of the material skeleton structure [18,19,20]. In addition, bimetallic MOFs-derived carbon materials possess more exposed active centers, good stability, and electrical conductivity, enabling them to have broader applications in electrochemical energy storage and conversion [21].

In this paper, the bimetallic MOF (CoCu-pPD) was synthesized from Co(NO_3_)_2_·6H_2_O, Cu(NO_3_)_2_·3H_2_O and pPD as raw materials, and N, O co-doped carbon material (BMHCS) was obtained by a simple pyrolysis process, showing the morphology of hollow spheres assembled by nanosheets. It has been found that the introduction of Cu^2+^ not only tunes the morphology but also enhances the framework stability of the main structure of Co-pPD. The BMHCS well inherits the hollow spherical morphology of the precursor and has a high specific surface area and heteroatom doping. When used as the anode material of SIBs, it shows a higher sodium storage capacity than Co-pPD-derived carbon nanosheets (MCNS) and exhibits high cycling performance (the capacity of 306 mA h g^−1^ after 300 cycles at the current density of 1 A g^−1^, with the cycle retention rate of 90%) and rate performance (the sodium storage capacity of 260 mA h g^−1^ at a high current of 5 A g^−1^). This is attributed to the fact that the hollow spherical structure assembled by nanosheets is beneficial to the stability of carbon materials during sodium storage, and the heteroatom doping, high specific surface area, and abundant pore structure can provide more surface defects and active sites for carbon materials, which increase sodium storage capacity.

## 2. Materials and Methods

### 2.1. Synthesis of MOFs and Derived Carbon Materials

Synthesis of MOFs: 1.75 g of Cobalt nitrate hexahydrate (Co(NO_3_)_2_·6H_2_O, Shanghai D&B Biological Science and Technology Company, China, 98%) and 0.65 g of p-phenylenediamine (pPD, Macklin, 99%) were dissolved in absolute ethanol (30 mL), respectively. After mixing the two reactants, 1.45 g of Cupric nitrate hexahydrate (Cu(NO_3_)_2_·3H_2_O, Beijing Tongguang Fine Chemical Company, China, 99.5%) was added and stirred uniformly. Then, it was loaded into a hydrothermal reactor for solvothermal reaction in a blast furnace (reaction at 150 °C for 24 h). After being cooled to room temperature, the precipitate was washed several times with ethanol and dried in a vacuum oven to obtain the final product, denoted as CoCu-pPD. Other experimental conditions remained unchanged, and the product obtained without adding Cu(NO_3_)_2_·3H_2_O was denoted as Co-pPD. This method is a common solvothermal method for preparing the MOFs. For example, Guan et al., used solvothermal method to grow bimetallic (NiCo) MOF on an NF surface [22].

Synthesis of the MOFs-derived carbon materials: The CoCu-pPD and Co-pPD were placed into a tubular carbonization furnace with N_2_ flow, and the temperature was increased from room temperature to 700 °C at a rate of 5 °C/min and kept for 2 h–denoted as CoCu/C and Co/C, respectively. The obtained carbonized products were acid-washed with concentrated HCl solution to obtain pure carbon materials, which were denoted as BMHCS and MCNS, respectively.

### 2.2. Analysis and Measures

The morphologies and internal structures of the MOFs and carbon materials were observed by scanning electron microscopy (SEM, Supra-55) and transmission electron microscopy (TEM, Hitachi HT-7700). The crystal structures of the MOFs and carbon materials were determined by X-ray diffraction (XRD, Rigaku D/max2500B2+/PCX system). The thermogravimetric test (TG, Netasch STA 449C) was used to investigate the structural changes of CoCu-pPD and Cu-pPD during pyrolysis and to compare the framework stability of the two MOFs. The specific surface area and pore size distribution of BMHCS and MCNS were obtained using a nitrogen adsorption and desorption test (ASAP 2020), which were based on the brunauer-emmett-teller (BET) model and nonlocal density functional theory (NLDFT), respectively. The defect sites and surface chemical elements of the BMHCS and MCNS were analyzed by Raman spectroscopy (Labram Aramis) and X-ray photoelectron spectroscopy (XPS, Escalab 250), respectively.

### 2.3. Electrochemical Measurements

The BMHCS or MCNS, acetylene black, and polyvinylidene fluoride were mixed in a weight ratio of 8:1:1, and an appropriate amount of N-methylpyrrolidone was dripped into the mixture to form a slurry. Subsequently, the slurry was coated on copper foil, dried in vacuum at 120 °C, and then cut into 12 mm circular electrode sheets. The SIBs were assembled using CR2025 cells in an argon-filled glove box (Unilab M Braun). Metal sodium was used as the negative electrode, glass fiber was used as the separator, and 1 M NaSO_3_CF_3_ dissolved in diglyme (1.0 M NaSO_3_CF_3_ in diglyme = 100 vol%) was used as the electrolyte. Cyclic voltammetry measurements were performed using an electrochemical workstation (Zennium, Zahner) at different scan rates (0.2, 0.5, 0.7, 1, 2, 5 mV/s) with a voltage range of 0.01–3 V. The galvanostatic charge–discharge tests were carried out by the blue electric test system (LAND-CT2001A) with a voltage range of 0.01–3 V (set the current density to 1 A g^−1^ for 300 cycles to test the electrochemical cycle performance, and set the current density to 0.05, 0.1, 0.2, 0.5, 1, 2, 5 A g^−1^ for 10 cycles to test the electrochemical magnification performance). The AC impedance measurements were performed using an electrochemical workstation (Zennium, Zahner, Germany) with an alternating voltage amplitude of 5 mV and a frequency range of 100 kHz to 10 mHz.

## 3. Results and Discussion

### 3.1. The Performance of the Co-pPD and CoCu-pPD

**Figure 1:**
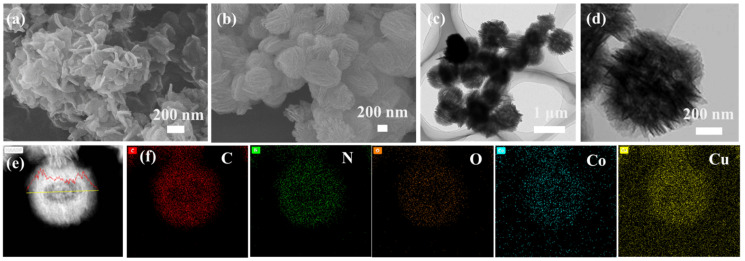
(**a**) SEM image of Co-pPD; (**b**) SEM image of CoCu-pPD; (**c**,**d**) TEM images of CoCu-pPD; (**e**) High-angle annular dark-field scanning transmission electron microscopy (HAADF-STEM) image of CoCu-pPD; (**f**) The corresponding elemental mapping of C, N, O, Co, and Cu elements on the CoCu-pPD.

Co-pPD exhibits the morphology of sheet-like random stacking (Figure 1a), while the bimetallic MOF (CoCu-pPD) formed by the coordination reaction of Co^2+^, Cu^2+^ and pPD exhibits spherical morphology assembled by nanosheets (the average particle size: 620 nm), as illustrated in Figure 1b–d, indicating that the addition of Cu^2+^ can regulate the morphology of MOFs. The line scan and mapping of the EDS of the carbon spectrum are shown in the Figure 1e,f, which show typical hollow structures. It is consistent with the change trend of line scan and mapping of carbon spectrum in hollow nanostructures mentioned in this article reported by Zhou et al. [23], so the hollow structure is further identified. The elemental mapping results in Figure 1f indicate uniform distributions of C, N, O, Co, and Cu elements on the CoCu-pPD, which provides the evidence that the structure and composition of the MOF are uniform.

**Figure 2:**
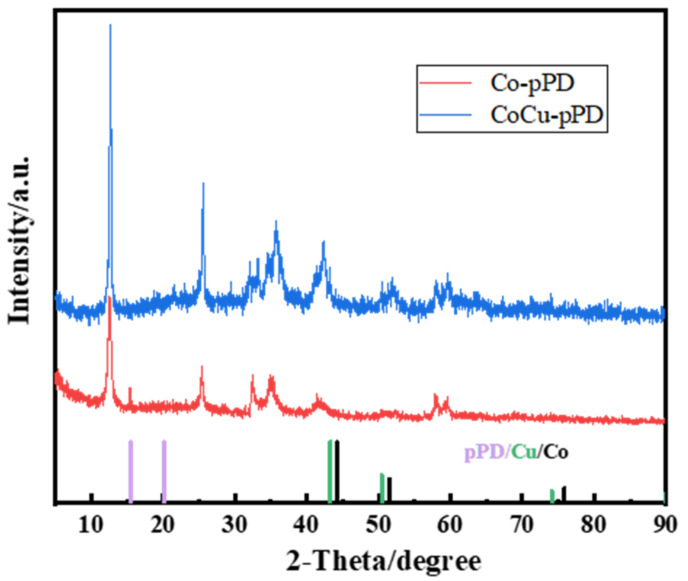
XRD patterns of Co-pPD and CoCu-pPD.

The crystal structures of the two MOFs were characterized by XRD. Both the XRD patterns of Co-pPD and CoCu-pPD show sharp peaks, which are different from the characteristic peak of pPD (PDF#31-1832), indicating that the two kinds of MOFs are highly crystalline (Figure 2). From the two spectra, although the framework structures are not identical, the main crystal structures are similar, and the addition of Cu^2+^ makes the crystallinity of the product MOFs higher. In addition, the multiple small peaks between 60° and 80° indicate that the coordination form of CoCu-pPD is more complex. In summary, the addition of Cu^2+^ can significantly change the degree of crystallization–as well as part of the coordination–but does not significantly change the crystal structure of the MOFs.

**Figure 3:**
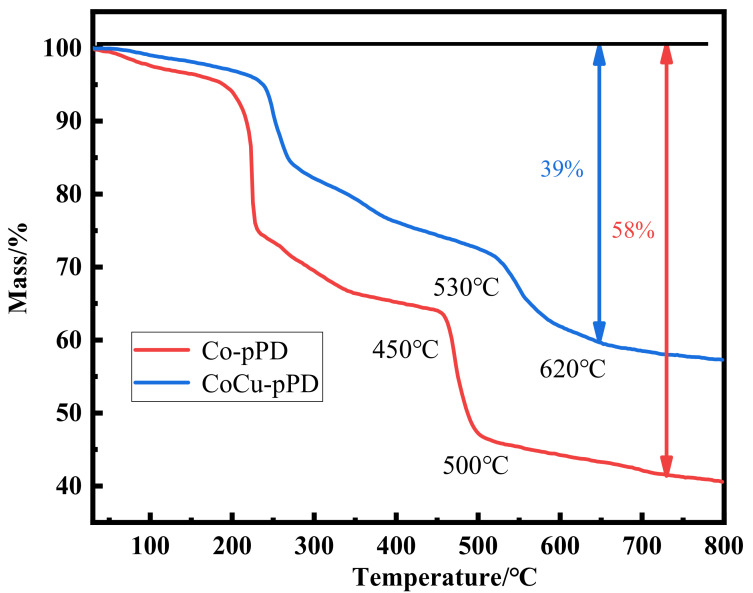
TG curves of Co-pPD and CoCu-pPD.

As shown in Figure 3, it can be seen from the TG curve that the weight loss of Co-pPD mainly goes through two stages. The mass loss in the first stage 250–300° is mainly the volatilization of water molecules and ethanol solvent molecules adsorbed in the MOFs structure during the preparation and placement process [24]. The temperature between 450–500 °C is the second stage, which is mainly caused by the decomposition process of the MOFs. The weight loss behavior of CoCu-pPD is similar to that of Co-pPD, but the decomposition temperature range in the second stage is between 530–620 °C, which is 100 °C higher than that of Co-pPD, indicating that the thermal stability of CoCu-pPD is better than that of Co-pPD. In addition, the final mass loss (39%) of CoCu-pPD is also smaller than that of Co-pPD (58%) under the same pyrolysis conditions. It is consistent with the results mentioned in many literatures that bimetallic MOFs can exhibit high thermal stability due to the synergistic effect of the two metals [25,26,27].

### 3.2. The Performance of MCNS and BMHCS

**Figure 4:**
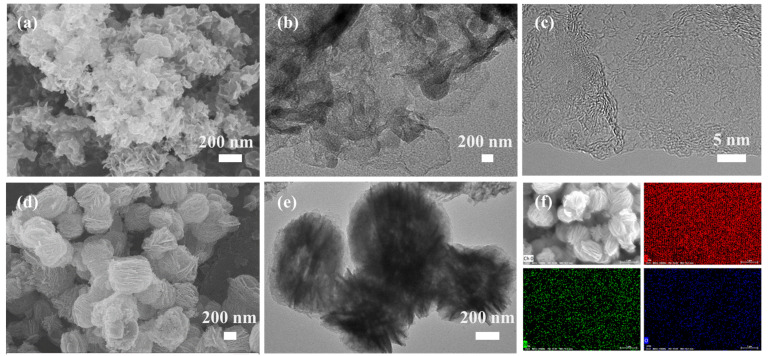
(**a**) SEM image of MCNS; (**b**) TEM image MCNS; (**c**) HRTEM images of MCNS; (**d**) SEM image of BMHCS; (**e**) TEM image of BMHCS; (**f**) The corresponding elemental mapping of C, N, O elements on the BMHCS.

Both MOFs can maintain the morphology after high-temperature carbonization (Co/C and CoCu/C), as showed in Figure S1. The Co-pPD-derived carbon nanosheets (MCNS) have a randomly stacked sheet-like morphology similar to Co-pPD, as illustrated in Figure 4a,b, and no obvious crystallites can be seen in the HAADF-STEM image (Figure 4c), indicating that the crystal structure of the precursor is destroyed. The SEM image (Figure 4d) shows that the CoCu-pPD-derived hollow carbon spheres (BMHCS) maintain the morphology of the precursor and is still displays spheres assembled from sheets (the average particle size: 550 nm). It is not only a sheet-like assembled ball, but also a hollow spherical structure, as can be recognized from the TEM image (Figure 4e). This means that CoCu-pPD has good framework stability and can still maintain the morphology and hollow structure of the precursor after carbonization and acid washing. In addition, the metal elements in BMHCS have been removed, and only three elements (C, N, O) are uniformly distributed on the surface in the sample (Figure 4f).

**Figure 5:**
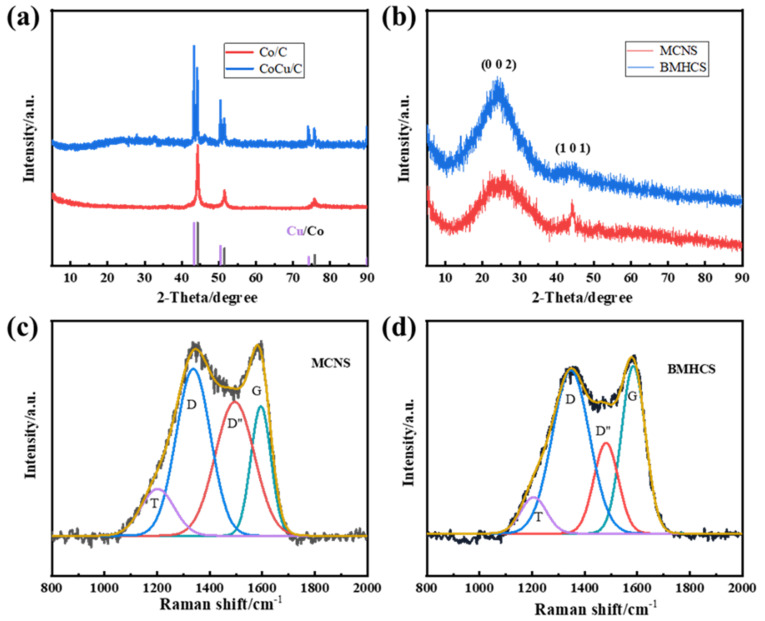
(**a**) XRD patterns of Co/C and CoCu/C; (**b**) XRD patterns of MCNS and BMHCS; (**c**) Raman spectra of MCNS; (**d**) Raman spectra of BMHCS.

The sharp peaks present at 44°, 52°, and 75° in the XRD pattern (Figure 5a) of Co/C (carbonized product of Co-pPD at 700 °C) correspond to the (111), (200), and (220) crystal planes of metallic Co (PDF#05-0727), respectively. In addition to metallic Co, the XRD pattern of the CoCu/C (Figure 5a) also shows the peak of metallic Cu (PDF#04-0836), indicating that CoCu-pPD contains both Co and Cu after carbonization. There are only two broad diffraction peaks at 25° and 43° in the XRD patterns of MCNS and BMHCS, as shown in Figure 5b, indicating that carbon materials obtained after metal removal by concentrated HCl are amorphous.

The Raman spectra of MCNS and BMHCS are displayed in Figure 5c,d, and they can be fitted into four peaks, in which the peaks at 1348 cm^−1^ and 1585 cm^−1^ correspond to D peak of amorphous carbon and G peak of graphite structure, respectively [28]. It is found that the I_D_ (peak area of D peak) of both carbon materials are larger than I_G_ (peak area of G peak) and the I_D_/I_G_ value of MCNS (2.21) is larger than that of BMHCS (1.51), indicating that MCNS is more amorphous and contains more defects than BMHCS. The defects of MCNS and BMHCS provide more sodium storage sites and facilitate the transmission of Na^+^ during charging and discharging.

The elements and contents of carbon materials were analyzed by the XPS test and the results are shown in Figure S2a,b and Table S1. In the survey spectra of MCNS and BMHCS, in addition to the C peak, there are also N and O peaks. There is little difference in the amount of N atoms contained in MCNS and BMHCS as displayed in Table S1, but the content of O atoms in BMHCS is less, which may be related to the adsorption of O-containing molecules during the preparation process. In order to further reveal the chemical state of each element of carbon materials, C1s, N1s, and O1s spectra are divided into peaks. The C1s spectra (Figure S2a_1_,b_1_) of the two carbon materials can be divided into C=C/C-C (284 eV), C-O/C-N (286 eV), and C=O (289 eV) peaks. The peaks around 398, 400, and 401 eV in the N1s spectrum (Figure S2a_2_,b_2_) represent N-6 (pyridine nitrogen), N-5 (pyrrole nitrogen), and N-Q (graphite nitrogen), respectively, and the peaks at 531, 532, and 534 eV in the O1s spectrum (Figure S2a_3_,b_3_) correspond to C=O, C-OH/C-O-C, and COOH, respectively [29]. The peak splitting results of the two carbon materials are the same, but the content of N-5 and N-6 of BMHCS is higher than that of MCNS (Table S1). N-5 and N-6 can make the adjacent C atoms as the active center, which is conducive to the adsorption of Na^+^, thereby improving the electrochemical performance of carbon materials [30,31]. In addition, oxygen-containing functional groups can also increase the interlayer spacing and defect sites of carbon materials, increasing the sodium storage capacity and cycle performance [32].

**Figure 6:**
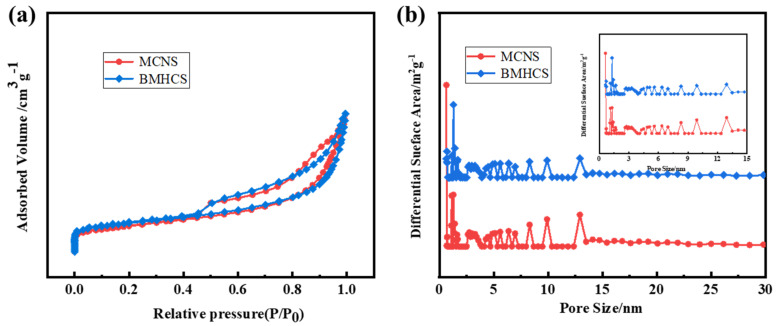
(**a**) N_2_ adsorption−desorption isotherms; (**b**) Pore width distribution curves of MCNS and BMHCS.

The N_2_ adsorption and desorption curves of MCNS and BMHCS (Figure 6a) are both IV isotherms, in which the rapidly increasing part of the adsorption amount at low relative pressure represents the existence of micropores, and the hysteresis ring represents the existence of mesopores [33,34]. As shown in Figure 6b, MCNS and BMHCS coexist with micropores and mesopores, and the pore size is mainly concentrated within 15 nm. The total pore volumes of two kinds of carbon materials are not very different–all close to 0.9 cm^3^ g^−1^–but the specific surface area of BMHCS (418 m^2^ g^−1^) is larger than that of MCNS (386 m^2^ g^−1^). The large specific surface area and pore structure can provide more active sites and more mass transfer channels for Na^+^ storage.

**Figure 7:**
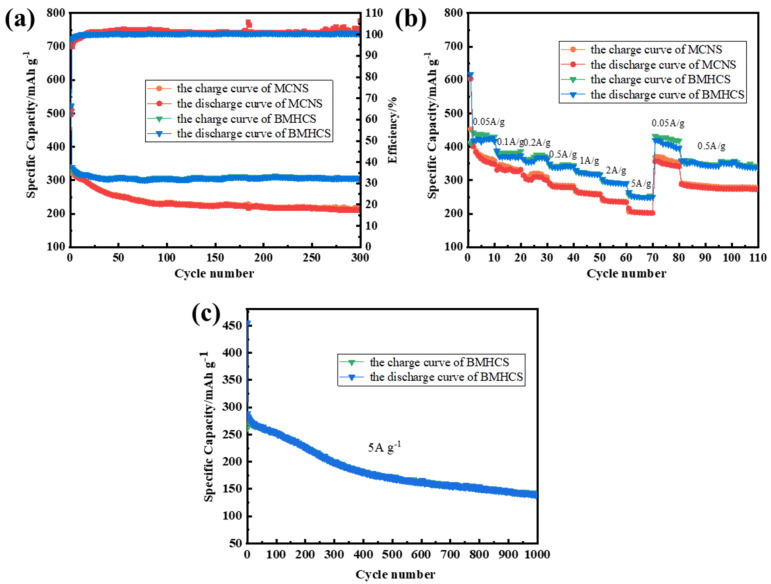
(**a**) Cycling performance of MCNS and BMHCS at 1 A g^−1^; (**b**) Rate performance of MCNS and BMHCS from 50 mA g^−1^ to 5 A g^−1^; (**c**) Cycling performance of BMHCS at 5 A g^−1^.

The cycle performance and rate performance of the carbon materials were tested (Figure 7a,b) when used as the anode material of SIBs. BMHCS exhibits higher first coulombic efficiency effect (507 mA h g^−1^, 68.22%) than MCNS (496 mA h g^−1^, 53, 5%) at a current density of 1 A g^−1^. The main reason is that BMHCS has a higher specific surface area and contains more N-5 and N-6, which can provide more sodium storage sites. After 300 cycles, the specific capacities of MCNS and BMHCS are 213 and 306 mAh g^−1^, with the capacity retention rate of 62.9% and 90.8% (Figure 7a). It is obvious that the cycle performance of BMHCS is also higher than that of MCNS, which is attributed to the unique sheet-like assembled hollow spherical structure of BMHCS that can adapt to the continuous de-intercalation of Na+ during the process of sodium storage and maintain the stability of the structure. As shown in Figure 7b, BMHCS exhibits a higher specific capacity than MCNS at different current densities, and still maintains the capacity of 260 mA h g^−1^ at a large current density of 5 A g^−1^, with the sodium storage capacity of 160 mA h g^−1^ after 1000 cycles (Figure 7c). In addition, BMHCS also possesses the capacity recovery rate of 90% when the current density recovers from 5 A g^−1^ to 0.5 A g^−1^, exhibiting excellent rate performance. Its electrochemical performance is higher than previously reported MOFs-derived pure carbon materials for the anode materials of SIBs, as displayed in Table 1. In addition, Table 1 also shows the performance of bimetallic MOFS-derived carbon materials for lithium ion batteries, which is a reference for scholars interested in bimetallic MOFS-derived carbon materials for lithium-ion batteries.

**Figure 8:**
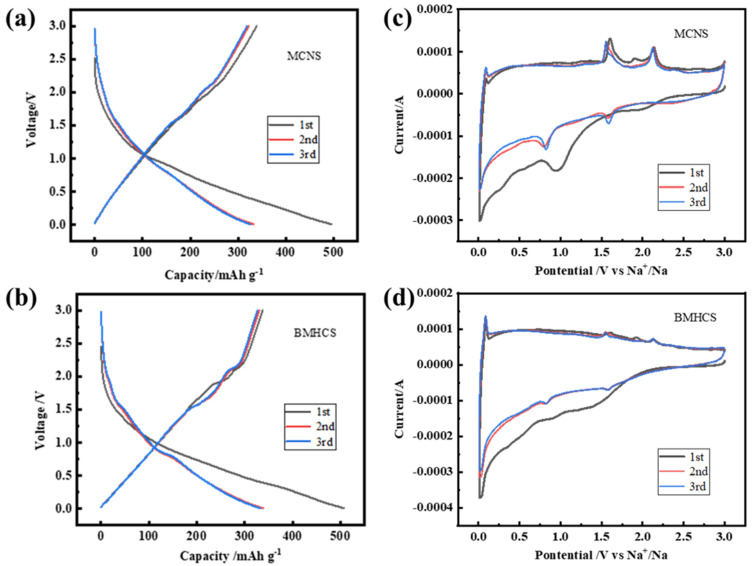
(**a**,**b**) First three charge–discharge curves at 1 A g^−1^; (**c**,**d**) CV curves at 0.2 mV s^−1^ of the initial three cycles of MCNS and BMHCS.

The first three charge–discharge curves of MCNS and BMHCS at a current density of 1 A g^−1^ are shown in Figure 8a,b, which show a sloping trend and no obvious plateau. The inclined area represents the insertion and extraction of Na^+^ at the surface defects, indicating the capacitive adsorption behavior of Na^+^ [9,42]. The first discharge curves are different from the others because of the formation of the SEI film during the first charge and discharge [43]. The first charge–discharge CV curves of MCNS and BMHCS at the scan rate of 0.2 mV s^−1^ (Figure 8c,d) show an irreversible reduction peak in the range of 1.5–0.5 V, which are caused by the formation of the SEI film and the irreversible consumption of Na^+^ during the first charge–discharge process [44]. The reduction peak around 0.01 V indicates that Na^+^ is mainly embedded in the carbon material in the voltage range around 0.01 V, and the oxidation peak near 0.1 V represents the desorption of Na^+^ from the carbon material [45].

The changing behavior of the CV curves of MCNS and BMHCS at different scan rates (0.5, 0.7, 1, 2, 5 mV s^−1^) are the same as those of 0.2 mV s^−1^, but the current increases with the increase in the scan rate and the oxidation peak gradually broadens with the increase in scan rate, indicating that the voltage range of Na^+^ extraction from carbon material becomes wider (Figure S3a,b). The relationship between the current I and the scan rate v obeys the law of I = av^b^ (a and b are adjustable parameter, b can be obtained from the slope of log(v)-log(i); usually the value of b between 0.5 and 1 represents pseudocapacitive properties) [46,47]. The b values of MCNS and BMHCS are all about 0.8 by analysis (Figure S3a_1_,b_1_), showing the high capacitive controlled during the process of sodium storage. Moreover, by analyzing the capacitive contributions under different scanning rates, as displayed in Figure S3a_2_,b_2_, the contribution rates of the capacitive capacity of the two kinds of carbon materials at the scanning rate of 0.2 mV s^−1^ are 58% and 59%, respectively, and with the increase in the scanning rate, there are higher capacitive contributions, which further indicates that MCNS and BMHCS mainly store sodium by the capacitive processes (Figure S3a_3_,b_3_).

The impedance spectra of MCNS and BMHCS and equivalent circuit diagram are shown in Figure S4a. According to the literature, R_f_ represents the surface diffusion resistance, and R_ct_ represents the charge transfer resistance [24]. According to the resistance value obtained by fitting (Figure S4b), it can be seen that the resistance values of R_f_ and R_ct_ of BMHCS are smaller than MCNS, indicating that it has more active sites, and the diffusion and charge transfer of Na^+^ are easier than that of MCNS during the charge and discharge process, so BMHCS shows better cycling performance and rate performance when is used for sodium storage.

## 4. Conclusions

In this work, the bimetallic MOF (CoCu-pPD) with pPD as the organic ligand was synthesized by a simple solution thermal method, and N/O co-doped sheet-like assembled hollow carbon spheres were prepared. The bimetal strategy not only modulates the MOF morphology, but also changes the disadvantage of poor thermal stability of MOFs with pPD as the ligand. The bimetallic MOFs-derived carbon material (BMHCS) exhibits excellent cycling and rate performance as the anode material for SIBs (306 mA h g^−1^ at 1 A g^−1^ after 300 cycles; 260 mA h g^−1^ at 5 A g^−1^) due to its unique hollow spherical structure, abundant defect sites, large specific surface area, and N/O co-doping. The morphology and framework stability of the MOFs are regulated by the introducing of Cu^2+^, which remarkable change the morphology, crystalline structure, specific surface area, and heteroatom doping of the derived carbon materials, improving their electrochemical performance.

## Figures and Tables

**Table 1:** Electrochemical performances of MOFs-derived carbon materials for the anode materials of SIBs and LIBs.

MOFs-Derived Carbon Materials	Cycle Performance	Rate Performance	Batteries
Hollow carbon spheres (this work)	306 mA h g^−1^ at 1 A g^−1^ after 300 cycles	260 mA h g^−1^ at 5 A g^−1^	SIBs
Cube-shaped porous carbon [35]	240 mA h g^−1^ at 0.1 A g^−1^ after 100 cycles	100 mA h g^−1^ at 3.2 A g^−1^	SIBs
Hollow carbon nanobubbles [36]	100 mA h g^−1^ at 10 A g^−1^ after 1000 cycles	100 mA h g^−1^ at 3.2 A g^−1^	SIBs
Hollow carbon nanobubbles [30]	236 mA h g^−1^ at 0.1 A g^−1^ after 100 cycles	142 mA h g^−1^ at 5 A g^−1^	SIBs
3D hollow porous carbon microspheres [37]	313 mA h g^−1^ at 0.1 A g^−1^ after 100 cycles	112.5 mA h g^−1^ at 5 A g^−1^	SIBs
Ni-doped Co/CoO/NC hybrid [38]	218 mA h g^−1^ at 0.05 A g^−1^ after 100 cycles	110 mA h g^−1^ at 5 A g^−1^	SIBs
ZnFe_2_O_4_@C nanocomposites [39]	1780 mA h g^−1^ at 1 A g^−1^ after 400 cycles	918 mA h g^−1^ at 3 A g^−1^	LIBs
Hollow Fe–Mn–O/C razmak microspheres [40]	1294 mA h g^−1^ at 0.1 A g^−1^ after 200 cycles	521 mA h g^−1^ at 1 A g^−1^	LIBs
Carbon-coated Cu-Co razmak bimetal oxide composite material [41]	900 mA h g^−1^ at 0.1 A g^−1^ after 100 cycles	507 mA h g^−1^ at 1 A g^−1^	LIBs

## Data Availability

Data are contained within the article or supplementary materials.

## Supplementary Materials

The following supporting information can be downloaded at: https://www.mdpi.com/article/10.3390/nano12213926/s1, Figure S1: (a) SEM image of Co/C; (b) SEM image of CoCu/C; Figure S2: (a) XPS survey spectrum; (a_1_) C1s spectra; (a_2_) N1s spectra; (a_3_) O1s spectra of MCNS; (b) XPS survey spectrum; (b_1_) C1s spectra; (b_2_) N1s spectra; (b_3_) O1s spectra of BMHCS; Table S1: Elemental composition of MCNS and BMHCS according to XPS test; Figure S3: (a,b) CV curves at different scanning rates; (a_1_,b_1_) The fitted linearity between redox peak current and the square root of the scan rate; (a_2_,b_2_) CV curve at 0.2 mV s^−1^, shaded part represents capacitive contribution; (a_3_,b_3_) Contribution rate of the capacitive and diffusion-controlled at different scanning rates of MCNS and BMHCS; Figure S4: (a) Impedance profiles; (b) Equivalent circuit diagram and parameters of MCNS and BMHCS.
