# Supplementary material for: Bimetallic MOFs-Derived Hollow Carbon Spheres Assembled by Sheets for Sodium-Ion Batteries
Source: Nanomaterials (Basel). 2022 Nov 7;12(21):3926. doi: 10.3390/nano12213926 (PMC9655117; doi:10.3390/nano12213926)
Supplement: Supplementary file 1 [file nanomaterials-12-03926-s001.zip › nanomaterials-1966682-supplementary.pdf]

nanomaterials

Supplementray Materials

Bimetallic MOFs-Derived Hollow Carbon Spheres Assembled by Sheets for Sodium-Ion Batteries

Hui Yang [1], Ang Li [1,*], Chunli Zhou [1,2], Xuewei Liu [1], Xiaohong Chen [1], Haiyan Liu [3], Tao Liu [3] and Huaihe Song [1,*]

1 State Key Laboratory of Chemical Resources Engineering, Beijing Key Laboratory of Electrochemical Process and Technology for Materials, Beijing University of Chemical Technology, Beijing 100029, China
2 Tangshan Key Laboratory of Optoelectronic Conversion Materials, School of Physical Science and Technology, Tangshan Normal University, Tangshan 063000, China
3 Shandong Energy Group Ltd., Zhoucheng 277527, China
* Correspondence: li_ang@mail.buct.edu.cn (A.L.); songhh@mail.buct.edu.cn (H.S.); Tel.: +86-10-64434916 (H.S.)

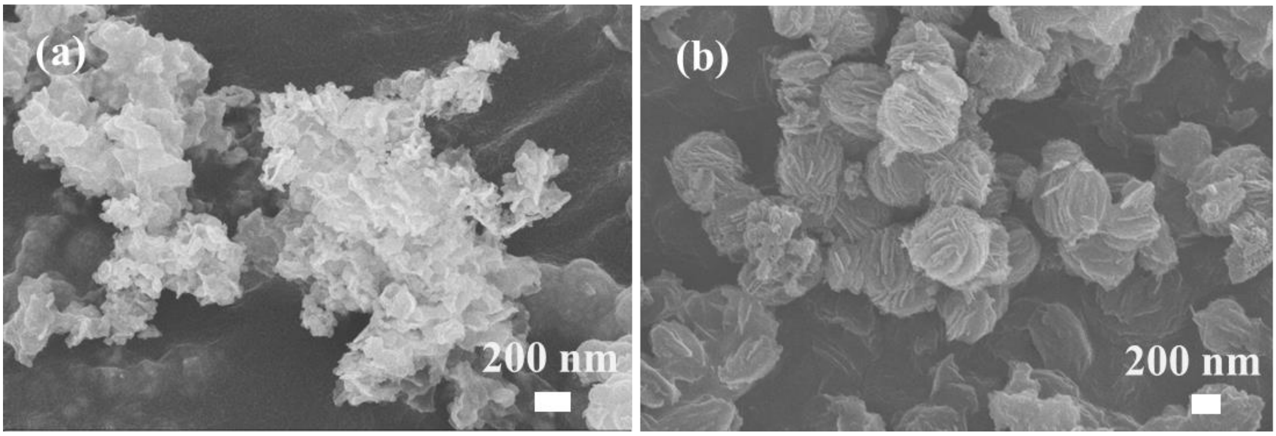

Figure S1. (a) SEM image of Co/C; (b) SEM image of CoCu/C.

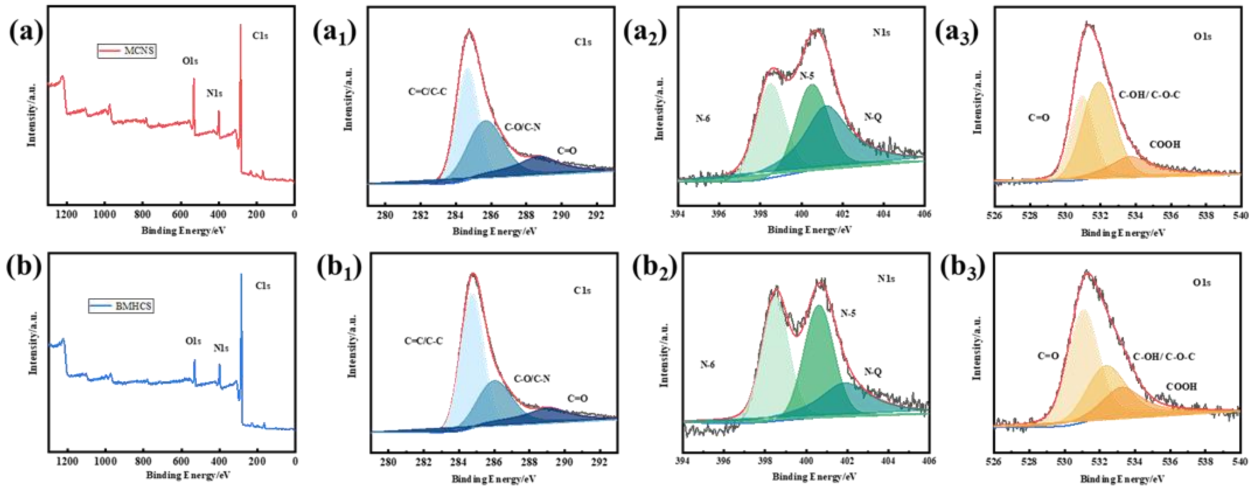

Figure S2. (a) XPS survey spectrum; (a$_1$) C1s spectra; (a$_2$) N1s spectra; (a$_3$) O1s spectra of MCNS; (b) XPS survey spectrum; (b$_1$) C1s spectra; (b$_2$) N1s spectra; (b$_3$) O1s spectra of BMHCS.

Table S1. Elemental composition of MCNS and BMHCS according to XPS test.

Sample	N /at%	N-5	N-6	N-Q	O /at%	C=O	C-OH/C-O-C	COOH
MCNS	9.72	3.04	2.86	3.82	11.25	3.74	5.63	1.88
BMHCS	10.04	3.89	3.69	2.46	6.17	3.18	1.85	1.14

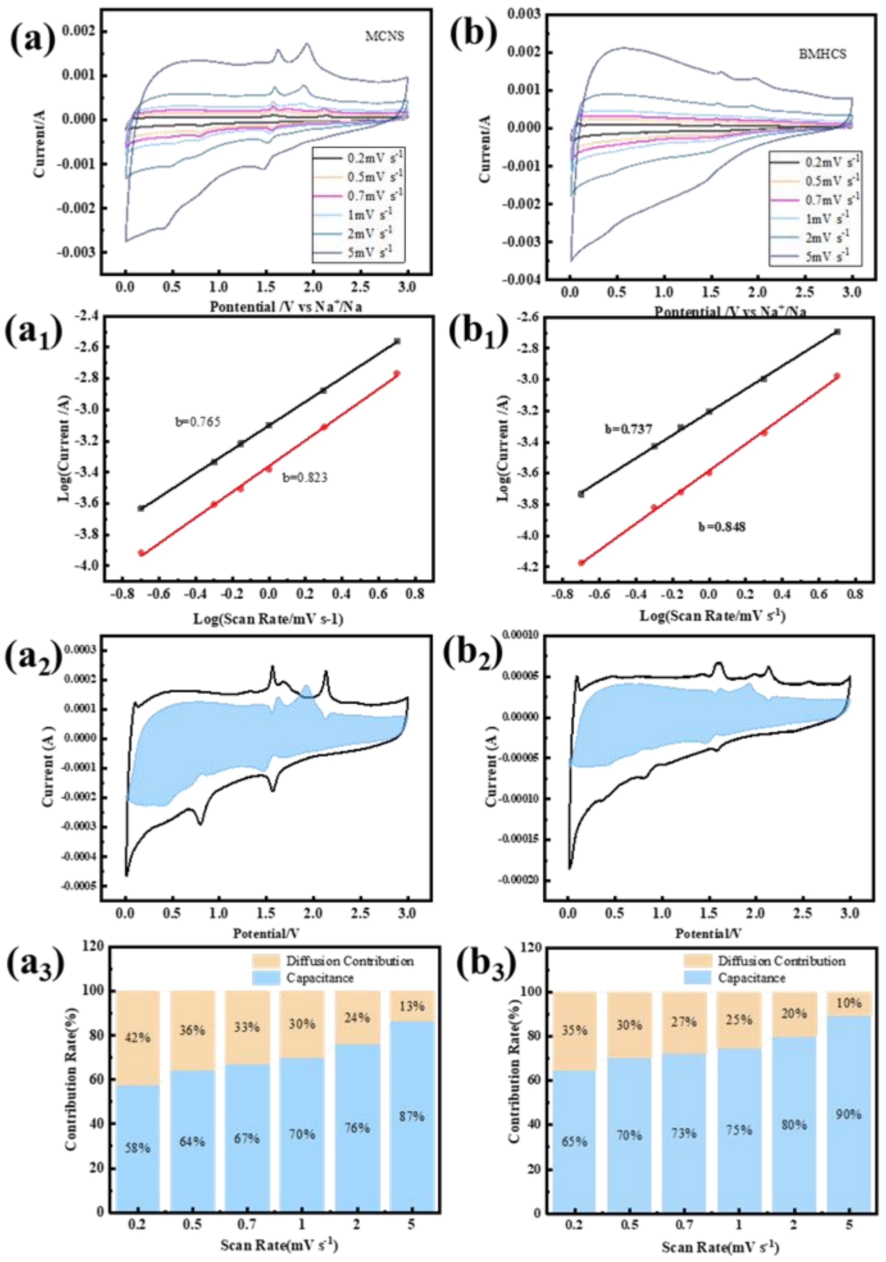

Figure S3. (a, b) CV curves at different scanning rates; (a_1, b_1) The fitted linearity between redox peak current and the square root of the scan rate; (a_2, b_2) CV curve at 0.2 mV s-1, shaded part represents capacitive contribution; (a_3, b_3) Contribution rate of the capacitive and diffusion-controlled at different scanning rates of MCNS and BMHCS.

(a)

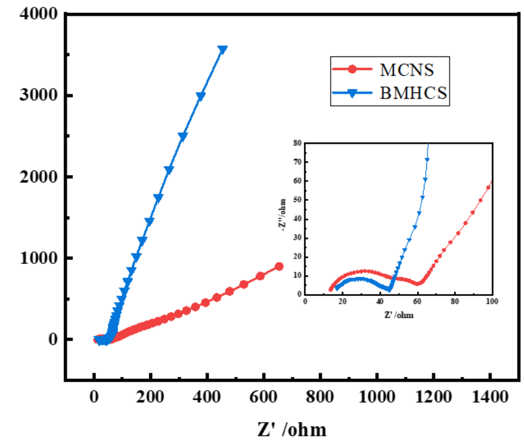

(b)

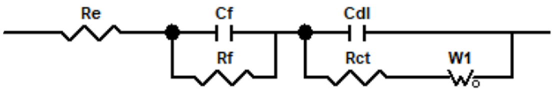

样品	Re	Rf	Rct
MCNS	14.25	22.75	18.85
BMHCS	16.53	10.61	13.04

Figure S4. (a) Impedance profiles; (b) Equivalent circuit diagram and parameters of MCNS and BMHCS.
